# Transcriptomic analysis reveals light quality response and systemic nutrient allocation under monochromatic light environments in *Nicotiana tabacum*

**DOI:** 10.1186/s12870-025-07974-w

**Published:** 2025-12-20

**Authors:** Jinge Du, Yiming Liu, Zhiqiang Xu, Xianwei Hao, Hairuo Song, Mengnan Guo, Yu Zhang, Lin Meng

**Affiliations:** 1 Key Laboratory of Tobacco Biology and Processing, Ministry of Agriculture, Tobacco Research Institute, Chinese Academy of Agricultural Sciences, Qingdao, 266101 PR China; 2 China Tobacco Zhejiang Industry Co., Ltd, Hangzhou, ZheJiang 310024 PR China; 3 China Agricultural University, Beijing, 100193 PR China

**Keywords:** Tobacco, Light quality, Nitrogen uptake, Potassium uptake, Transcription factor

## Abstract

Light quality plays a crucial role in plant growth and development. Nevertheless, mechanistic insights regarding the effects of monochromatic red light and monochromatic blue light on the development of tobacco seedlings remain limited. This study aims to elucidate how monochromatic red and blue light specifically affect the growth of tobacco seedlings, with a focus on systemic nutrient allocation from roots. By analyzing morphological, physiological and transcriptomic changes in both shoots and roots, we aim to fill the gaps in our understanding of the interaction between light quality and nutrient absorption mechanisms. Physiological results indicated that red light promoted seedling growth and enhanced nitrogen (N)and potassium (K) uptake, whereas blue light exerted the opposite effect by inhibiting growth and reducing nutrient uptake. Transcriptome analysis revealed that genes related to N uptake (e.g., *NRT1.1* was induced by red light and suppressed by blue light), N assimilation and K uptake were significantly regulated by monochromatic light in roots. Furthermore, various transcription factors (TFs) involved in phytohormone signal transduction were significantly regulated by blue and red light. These findings suggest that red and blue light may modulate phytohormone metabolism through TF–mediated pathways in tobacco roots. Overall, this study provides valuable insights into the effects of light quality on plant growth and underscores the importance of elucidating light-mediated mechanisms for improving crop yield and quality.

**Supplementary Information:**

The online version contains supplementary material available at 10.1186/s12870-025-07974-w.

## Introduction

Plant growth is shaped by light together with other environmental factors such as temperature and humidity. In addition to determining photosynthetic efficiency, specific wavebands within the spectrum strongly modulate photomorphogenesis, secondary metabolism, and phytohormone biosynthesis [1–3]. The solar spectrum spans red, green, blue, far-red, and ultraviolet regions. Red and blue light, in particular, have been interrogated extensively because they are efficiently absorbed by photosynthetic pigments. Distinct photoreceptors perceive these monochromatic signals: phytochromes sense red light, whereas cryptochromes and phototropins sense blue light [4]. Upon light perception, the receptors interact with the E3 ubiquitin ligase CONSTITUTIVE PHOTOMORPHOGENIC 1 (COP1), a central repressor of photomorphogenesis [5]. COP1 was first shown to directly interact with HY5 through its WD40 domain and mediate HY5 degradation in darkness via the ubiquitin-proteasome system [6]. Subsequently, a batch of light-signaling transcription factors were identified as COP1’s targets such as BBX21, BBX24, and LAF1 [7–10]. Light also crosstalks with endogenous hormones, thereby coordinating plant growth. Several classical phytohormones—including ethylene (ETH), abscisic acid (ABA), gibberellins (GAs), cytokinins (CKs), jasmonates (JAs), and salicylic acid (SA)—respond to light quality [11, 12]. This reciprocal regulation weaves a complex light–hormone transcriptional network [13].

The absorption characteristics of primary photosynthetic pigments—chlorophylls and carotenoids—together with their associated photoreceptors determine the wavelength-dependent efficiency of both photosynthesis and photomorphogenesis [14]. In chicory, for example, the CO₂-assimilation rate is significantly lower under blue light than under red light [14]. Red light accelerates fruit ripening and carotenoid biosynthesis, whereas blue light inhibits hypocotyl elongation and promotes the accumulation of cytokinins and anthocyanins [15–17]. The *cryptochrome1/cryptochrome2 (cry1 cry2)* mutant, defective in both cryptochrome 1 and cryptochrome 2, has been widely used to dissect blue- and red-light signalling pathways [17]. Nevertheless, most studies that address spectral quality have been performed with plants cultivated under monochromatic light. Closed-environment plant-production systems in modern agriculture now allow precise control of light, CO_2_ concentration, nutrition, and water status.

Light-emitting diodes (LEDs) have emerged as the preferred illumination source because of their narrow emission spectra, long operational lifetime, low energy demand, and compact architecture. By selecting specific LED combinations, growers can tailor the spectral output to desired wave-bands, thereby increasing yield and improving quality traits in diverse crops [1]. Yorio et al. reported enhanced biomass in several vegetable species when monochromatic red light was supplemented with blue light [18]. Johkan et al. demonstrated that blue-LED irradiation of red-leaf lettuce seedlings improved seedling vigour and post-transplant performance [19]. Similarly, Samkumar et al. achieved a marked increase in bilberry anthocyanin content by providing supplemental red and blue light [20]. Collectively, these findings underscore the necessity of evaluating monochromatic-light effects on plant growth and development through integrated morphological, physiological, and biochemical analyses. The effects of LED light quality have also been studied in tobacco. Tran et al. applied LEDs in their study and found that under green and blue light, the resistance of tobacco plants to *Pseudomonas syringae pv. Tomato* was significantly higher than that under white and red light [21]. Maity et al. showed that red and blue LEDs with a ratio of 1:1 remarkably enhance the in vitro flowering of tobacco [22]. However, how LED light application affects the physiology and transcription of tobacco seedlings is still unclear.

Previous work has shown that red and blue light significantly influence the abundance of photosynthetic pigments, leaf CO_2_-assimilation rates, stomatal conductance, and the production of secondary metabolites such as phenylpropanoids, flavonoids, and anthocyanins [14] At present, the vast majority of studies have focused on above-ground organs—leaves, stems, fruits, and sprouts [11, 12, 14, 20, 23]—whereas the influence of monochromatic light on root development, in particular on root architecture, biomass, and phytohormone content, remains largely unexplored [8, 24]. Roots are essential vegetative organs that acquire the mineral nutrients required for plant growth; among these, nitrogen (N) and potassium (K) are predominant. Nitrogen is absorbed as nitrate (NO_3_⁻) or ammonium (NH_4_^+^) and subsequently assimilated via the nitrogen-metabolism pathway [25], whereas potassium is taken up by K⁺-transport proteins [26]. However, little is known about how monochromatic light affects nutrient acquisition by roots.

To elucidate how monochromatic red and monochromatic blue light specifically affect early-stage growth of tobacco seedlings—with emphasis on systemic nutrient allocation from roots—we simultaneously profiled morphological, physiological, and transcriptomic responses in both shoots and roots. Our aim is to address the gaps in understanding the relationship between light quality and nutrient absorption mechanisms. Through integrating transcriptional data with physiological measurements, we have investigated the effects of monochromatic light on N metabolism and K absorption, while also analyzing the role of transcriptional regulation in light signaling. The results provide a theoretical foundation for the optimal utilization of monochromatic light to enhance crop yield and quality.

## Materials and methods

### Plant materials and light treatments

Common tobacco (*Nicotiana tabacum L.*) cultivar “Zhongchuan208 (ZC208)”, obtained from the Tobacco Genetics and Breeding Research (Northern) Center of China (Qingdao), was planted in trays filled with peat and vermiculite in a greenhouse under natural illumination (approx. 800–1200 µmol·m^− 2^·s^− 1^ photosynthetic photon flux density, 14 h/10 h day/night cycle) with temperatures maintained at 28℃ (day) and 22℃ (night). Twenty-five days after germination, uniformly sized tobacco seedlings were transplanted to a liquid medium (1/4 Hoagland’s nutrient solution) and used for monochromatic light treatments. The pH of the solution was adjusted to 5.8 daily. The solution was fully replaced every three days, which adequately guaranteed oxygen availability for root ion absorption and eliminated the need for additional aeration. The seedlings were transferred to three chambers covered with photo-reflective sheets and equipped with top-mounted light-emitting diodes (LEDs), which generated white light, monochromatic blue light (peak wavelength: 448 nm), and monochromatic red light (peak wavelength: 660 nm), respectively. White light served as the control. All treatments were conducted under a 16 h light/8 h dark photoperiod, with daytime temperatures of 28 °C and nighttime temperatures of 22 °C. The dark period lasted from 10:00 PM to 6:00 AM the following day, during which the seedlings were grown in complete darkness. The light period ran from 6:00 AM to 10:00 PM, during which the seedlings were grown under red, blue, or white light. The above-canopy irradiance was maintained at 108 W/m^2^. Five biological replicates were set up for each light treatment. Leaves and roots of the tobacco seedlings were separately harvested at 9:00 AM on the seventh day of light treatment. The samples were immediately frozen in liquid nitrogen and subsequently stored at -80 °C for subsequent analysis. The LEDs were purchased from Qingdao Cale Photoelectric Technology Co., Ltd., Qingdao, China.

### Plant physiological analysis

Plant samples were dried at 70 °C for 72 h and weighed using a precision electronic balance (accuracy: 0.001 g) to determine dry weight (DW). To measure leaf area, the largest leaf from each tobacco seedling was selected, scanned at a consistent resolution, and its area was determined using Image J software. Root surface area was determined using the root analysis system WinRhizo (Regent Instruments, Montreal, QC, Canada), while the length of primary lateral roots was measured with a ruler and the numbers of primary and secondary lateral roots were counted; the average length of secondary lateral roots was calculated as (total root length − total length of primary lateral roots) / number of secondary lateral roots.

### Determination of chlorophyll content

Clean and dry leaves of tobacco seedlings were used. The leaf veins were removed, and the leaves were cut into approximately 1 cm × 1 cm pieces and mixed thoroughly. Ten milliliters of 95% ethanol was added, followed by soaking in the dark for 24 h with 2–3 shakes during the period to ensure full infiltration of the sample. After the sample tissue turned completely white, the supernatant was collected. Using 95% ethanol as the blank control, the absorbance was measured at 665 nm and 649 nm wavelengths (UV-1800, AOE Instruments Co., Ltd., Shanghai, China), respectively. Chlorophyll content was calculated using the Arnon formula: Chlorophyll a content (mg·g^− 1^) = (13.95×A665-6.88×A649)×V/(W×1000); Chlorophyll b content (mg·g^− 1^) = (24.96×A649-7.32×A665)×V/(W×1000). Wherein, “V” represents the volume of the extract (mL), and “W” represents the fresh weight of the sample (g). All analytical grade reagents were purchased from Shanghai Hushi Chemical Reagents Co., Ltd. (Shanghai, China).

### Measurement of total N and K content

Nitrogen (N) and potassium (K) contents were determined according to the method described by [27]. In short, the desiccated plant samples were ground into powder; about 50 mg of the powder was digested by 5 ml of 98% H_2_SO_4_ and 1 ml of 30% H_2_O_2_ at 270 °C; after cooling, the digested sample was diluted to 100 mL with distilled water. All reagents were purchased from Shanghai Hushi Chemical Reagents Co., Ltd. (Shanghai, China). Potassium ion (K⁺) and total N concentrations were measured using an Optima 2100 DV inductively coupled plasma optical emission spectrometer (ICP-OES; PerkinElmer, USA) and an AutoAnalyser 3-AA3 continuous flow chemical analyzer (Bran + Luebbe, Norderstedt, Germany), respectively. For K⁺ detection with ICP - OES: RF power is 1300–1500 W, plasma gas (argon) flow 13–15 L·min^− 1^, auxiliary gas (argon) flow 0.2–1.0 L·min^− 1^, nebulizer gas flow 0.7–1.0 L·min^− 1^, uses axial viewing mode, analyzes K⁺ at 766.491 nm (primary) and 769.896 nm (secondary), sample uptake rate ~ 1.5 mL·min^− 1^, integration time 3–5 s, with µg·L^− 1^ level detection limit. For total N detection with AutoAnalyser 3 - AA3 continuous flow chemical analyzer: It oxidizes N to NO_3_⁻ using alkaline potassium persulfate at ~ 120 °C for ~ 30 min, detects via colorimetry (540 nm, pink azo dye) or UV (220 nm, 275 nm correction), has reagent flow rate 1–3 mL/min with air - segmented flow, and ~ 0.1 mg·L^− 1^ detection limit.

### Measurement of plasma membrane H^+^-ATPase activity

Fresh plant samples were crushed, and impurities were removed through filtration and centrifugation to obtain purified plasma membranes [28]. The activity of plasma membrane (PM) H^+^-ATPase was determined by quantifying the amount of inorganic phosphorus released during the reaction [29]. In short, 30 µL of purified plasma membranes suspension was incorporated into the 0.5 mL reaction system (30 mM BTP/MES, 5 mM MgSO_4_, 50 mM KCl, 50 mM KNO_3_, 1 mM Na_2_MoO_4_, 1 mM NaN_3_, 0.02% (w/v) Brij 58, and 5 mM disodium-ATP [substrate]), incubated at 30 °C for 30 min, then terminated with 1 mL of a mixture (2% [v/v] H_2_SO_4_, 5% [w/v] SDS, 0.7% [w/v] (NH_4_)_2_MoO_4_) followed by 50 µL 10% (w/v) ascorbic acid. Ten minutes later, 1.45 mL of arsenite-citrate reagent (2% [w/v] sodium citrate, 2% [w/v] sodium arsenite, 2% [v/v] glacial acetic acid) was added, and after 30 min of color development, absorbance was measured at 720 nm. Activity—corrected against boiled-membrane controls—was expressed as phosphate liberated per g of membrane protein per h. All analytical grade reagents were purchased from Shanghai Hushi Chemical Reagents Co., Ltd. (Shanghai, China). All biological reagents used were purchased from Sigma-Aldrich Co. LLC. (St. Louis, MO, USA), while H_2_SO_4_ and glacial acetic acid were obtained from Shanghai Hushi Chemical Reagents Co., Ltd. (Shanghai, China).

### cDNA library preparation and transcriptome sequencing

Tobacco tissues from different light treatments were harvested for RNA sequencing (RNA-seq). Each sample had three biological replicates. RNA isolation and cDNA library construction were performed according to [30]. Total RNA was isolated using TRIzol reagent (Thermo Fisher Scientific, USA; Cat. No. 15596018). cDNA libraries were constructed using the TruSeq RNA Sample Preparation Kit (Illumina, USA; RS-930-2001). Transcriptome sequencing was carried out by Annoroad Gene Technology Co., Ltd. (Beijing, China) on an Illumina HiSeq 2500 platform with the 150 bp paired-end (PE) sequencing strategy.

### RNA-seq data processing and transcriptome assembly

Low-quality bases, adapter sequences, and reads shorter than 40 bp were first removed with Trimmomatic v0.36 following the developer’s guidelines [31]. STAR v2.7.8a software [32] was used to align the cleaned reads to the tobacco reference genome [33]. Transcript abundance was quantified and normalized with cuffquant and cuffnorm [34] and gene expression was reported as fragments per kilobase of transcript per million mapped fragments (FPKM). Genes with FPKM < 1 in all libraries were excluded from downstream statistical analyses.

### RNA isolation and RT-qPCR assay

Roots RNAs were extracted following the treatment with monochromatic red and blue light, as detailed in Sect. 2.1. Approximately 100 mg of root tissue sample was grinded into powder and subjected to TRIzol reagent (Thermo Fisher Scientific, USA; Cat. No. 15596018). cDNAs were using generated via HiScript II Q RT SuperMix kit with gDNA Wiper (Vazyme, Nanjing, China) following the manufacturer’s instructions. RT-qPCR analysis were detected using ChamQ SYBR Color qPCR Master Mix (Vazyme) on a StepOne Plus Real-Time PCR system (Applied Biosystems, Waltham, MA, USA). The tobacco ribosomal protein-coding gene *NtL25* (GenBank: L18908.1) [35] was used as a standard control. The relative transcript abundance was calculated using the 2^−ΔΔCT^ method. The data were presented as means ± se from 3 replicates, significance analysis was conducted using the Student’s *t*-test. The primers used are listed in Table S1.

### Statistical analysis

Differentially expressed genes (DEGs) were identified with DESeq2 using false-discovery rate (FDR) < 0.05 and |log_2_(fold change)| > 1 as thresholds [36]. Gene Ontology (GO) enrichment was performed with the agriGO v2.0 toolkit [37] at FDR < 0.05, and Kyoto Encyclopedia of Genes and Genomes (KEGG) pathway analysis was carried out with KOBAS 2.0 [38]. Principal-component analysis (PCA), volcano plots, heat maps, and hierarchical clustering dendrograms were generated with R software.

## Results

### Light quality regulates tobacco seedling growth and nutrient absorption

**Fig. 1:**
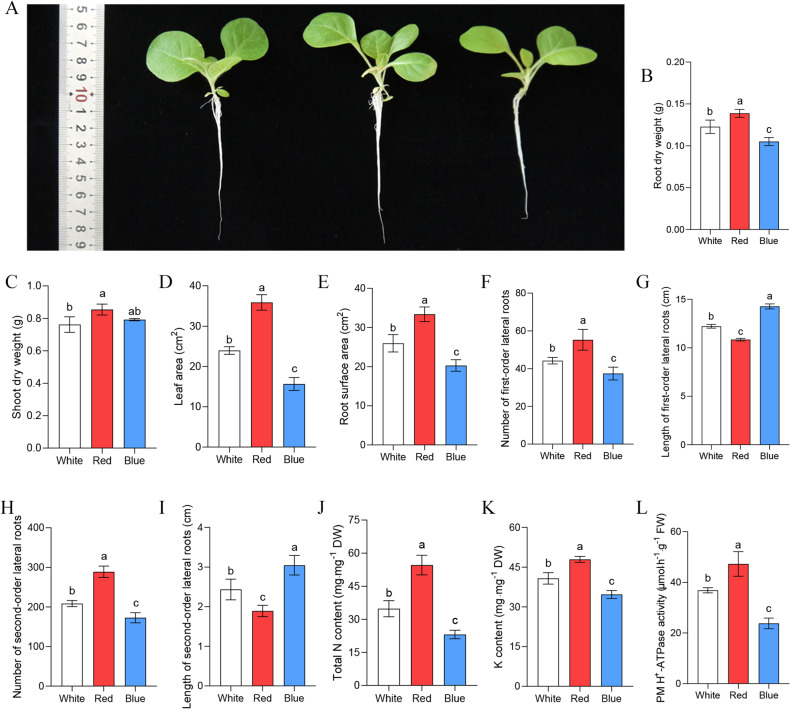
Physiological responses of tobacco seedlings to monochromatic light treatments. **A** Morphology of tobacco seedlings grown under different light quality. **B**,** C** Biomass of root **B** and shoot **C** of tobacco seedlings treated with different light quality. **D**,** E** Leaf area **D** and root surface area **E** of tobacco seedlings treated with different light quality. **F-I** First-order and second-order lateral root number and length in response to different light qualities. **J-L** Total N content **J**, K content **K** and PM H^+^-ATPase **L** in activity of roots under different light conditions. *N* ≥ 3, biological repetition. Different letters indicate significant differences between the data. Ordinary one-way ANOVA Tukey’s multiple comparisons test, *p* < 0.05

Light quality exerted a profound effect on the growth of tobacco seedlings (Fig. 1A).

Compared with the white light control, red light significantly increased root dry weight, whereas blue light reduced it (Fig. 1B). Above-ground biomass also responded spectrally: red light elevated shoot dry weight above control levels, but blue light elicited no significant change (Fig. 1C). Compared with the white-light control, red light significantly increased the leaf area of tobacco seedlings, while blue light exerted an inhibitory effect on leaf growth (Fig. 1D). In contrast, leaf number remained invariant across all treatments (Fig. S1A). Root nutritional status and plasma-membrane (PM) H⁺-ATPase activity were equally wavelength-dependent. As an important physiological indicator for assessing plant root absorption function, root surface area of tobacco seedlings was larger under red light and smaller under blue light (Fig. 1E). Compared with the white light control, red light increased the number of first and secondary lateral roots but reduced their length, whereas blue light exerted the opposite effects (Fig. 1F-I). Total nitrogen (N) and potassium (K) contents rose under red light and declined under blue light (Fig. 1J-K). Concordantly, PM H⁺-ATPase activity was upregulated by red light and downregulated by blue light (Fig. 1L). However, both red and white light suppressed chlorophyll accumulation in tobacco seedlings, with blue light exerting the strongest inhibitory effect (Fig. S1B, C).

### Light quality reshapes the tobacco seedling transcriptome

**Fig. 2:**
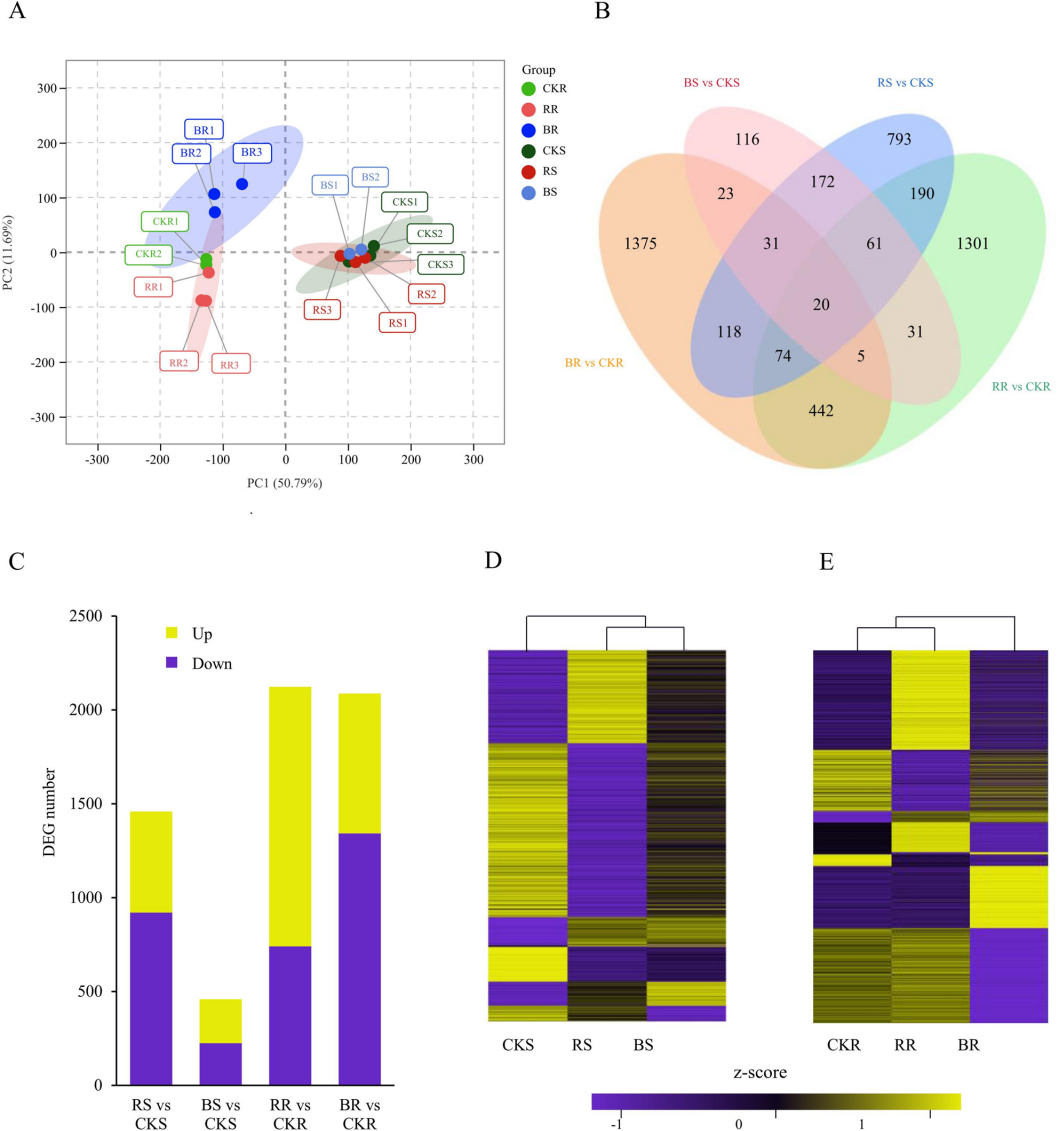
Identification and analysis of DEGs under different light treatments.** A** PCA analysis of transcriptome data obtained from shoots and roots of tobacco seedlings under different light treatments. **B** Venn diagrams showing DEGs in different tissues under different light treatments. **C** Quantification of DEGs in different tissues under different light treatments. **D**,** E** Heatmaps depicting the expression levels of DEGs in shoots (**D**) and roots (**E**). The dendrogram is presented at the top. *Abbreviations*: *CKS* Shoots under white light treatment, *RS* Shoots under monochromatic red light treatment, *BS* Shoots under monochromatic blue light treatment, *CKR* Roots under white light treatment, *RR* Roots under monochromatic red light treatment, *BR* roots under monochromatic blue light treatment

Tobacco seedlings were planted in monochromatic red light, monochromatic blue light and white light environments, respectively. Shoots and roots were collected separately for RNA sequencing. The raw sequencing data of multiple samples shows high-quality metrics (Q20% ≥ 96.36%, Q30% ≥ 91.44%) with consistent read length and sufficient clean data, supporting subsequent bioinformatics analyses (Table S2). After filtering of genes with low expression levels (FPKM < 1 in all samples), a total of 29,094 genes were retained for analysis. Principal component analysis (PCA) analysis of all these genes revealed two distinct groups corresponding to shoot and root samples, respectively (Fig. 2A). The first component (PC1; 62.57% variance explained) clearly separated the root from shoot samples, while the second component (PC2; 15.48% variance explained) discriminated among different light qualities. Pearson correlation matrix heatmap further confirmed distinct clustering patterns corresponding to different treatments (Fig. S2). Differentially expressed genes (DEGs) were identified between monochromatic lights and white light treatments in both shoots and roots (Table S3). Compared to the shoots under white light treatment (CKS), 1459 and 459 DEGs were identified in shoots under monochromatic red light treatment (RS) and monochromatic blue (BS) light treatment, respectively (Fig. 2B). Compared to roots under white light treatment (CKR), 2124 and 2088 DEGs were identified in roots under monochromatic red light treatment (RR) and monochromatic blue (BR) light treatment, respectively (Fig. 2B). A total of 284 DEGs were common to RS and BS, while 541 DEGs were common to RR and BR (Fig. 2B). Compared with the CKS, 538 and 233 DEGs were upregulated in RS and BS respectively, whereas 921 and 226 genes were downregulated in RS and BS, respectively (Fig. 2C). Compared with the CKR, 1382 and 745 genes were upregulated in RR and BR, respectively, with 742 and 1343 genes downregulated in RR and BR, respectively (Fig. 2C). The transcriptomic data were further subjected to hierarchical clustering analysis (Fig. 2D, E). Roots and shoots exhibited distinct dendrograms. For shoots, two distinct groups corresponded to the control and monochromatic light treatments, respectively (Fig. 2D). For roots, two distinct groups were identified: one corresponding to the red light and control treatments, and the other to the blue light treatment (Fig. 2E).

### GO and KEGG analysis

**Fig. 3:**
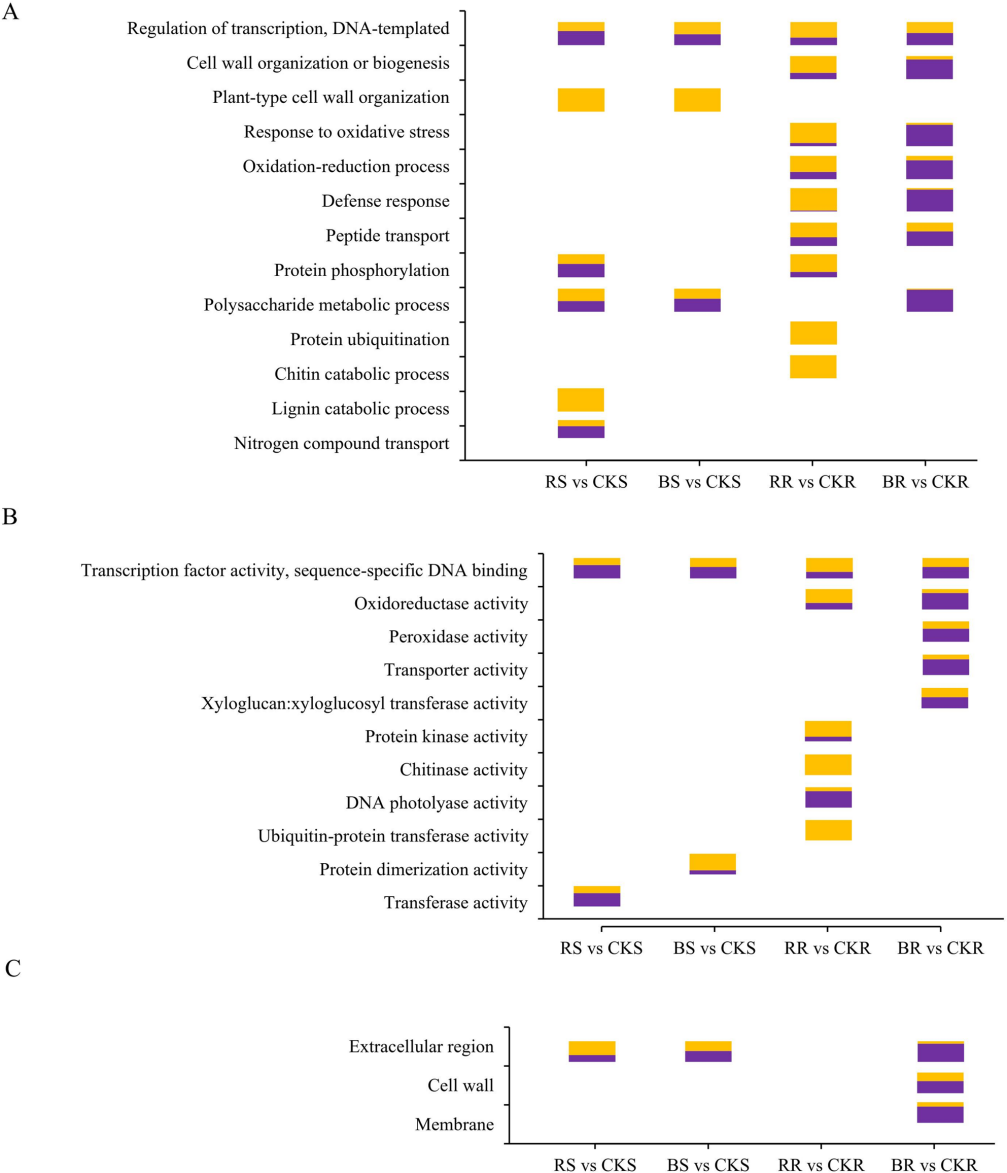
Most representative GO terms enriched in DEGs. **A-C** Biological process (**A**), molecular function (**B**), cellular component (**C**). Orange represents the percentage of upregulated genes, while purple represents the percentage of downregulated genes. *Abbreviations*: *CKS* Shoots under white light treatment, *RS* Shoots under monochromatic red light treatment, *BS* Shoots under monochromatic blue light treatment, *CKR* Roots under white light treatment, *RR* Roots under monochromatic red light treatment, *BR* Roots under monochromatic blue light treatment

GO analysis revealed that numerous GO terms were significantly enriched in the DEGs of RR/CKR, BR/CKR, RS/CKS, and BS/CKS (Table S4). Regarding biological processes, the GO term “transcription factor activity, sequence-specific DNA binding” was commonly enriched across all four tissue comparisons (Fig. 3A). Root-specific enriched terms (RR and BR) included “defense response”, “oligopeptide transport”, “oxidation-reduction process”, and “response to oxidative stress”, whereas “protein phosphorylation” was unique to red light treatments (RS and RR). The terms “protein ubiquitination” and “chitin catabolic process” were over-represented exclusively in RR, while “lignin catabolic process” and “nitrogen compound transport” were uniquely enriched in RS. Regarding molecular functions, “transcription factor activity, sequence-specific DNA binding” was the only GO term enriched in all four tissue comparisons (Fig. 3B). “Peroxidase activity”, “transporter activity”, and “xyloglucan: xyloglucosyl transferase activity” were enriched in BR, while “protein kinase activity”, “chitinase activity”, “DNA photolyase activity”, and “ubiquitin-protein transferase activity” were prevalent in RR. Furthermore, “protein dimerization activity” and “transferase activity” were enriched in BS and RS, respectively, whereas “oxidoreductase activity” was over-represented in both RR and BR. Regarding cellular components, DEGs in BR were significantly enriched in “extracellular region”, “cell wall”, and “membrane”, while DEGs in both RS and BS were mainly enriched in “extracellular region” (Fig. 3C).

GO analysis results indicated that transcriptional regulation played a central role in the response of both roots and shoots to monochromatic light. Monochromatic light treatment induced diverse physiological responses in roots, including oxidative stress response, defense response, altered transport activity, and oxidoreductase activity. Notably, numerous genes associated with “transport activity” were significantly regulated in BR, with the majority (81 out of 105) being downregulated (Table S4A, Fig. 3B).

**Fig. 4:**
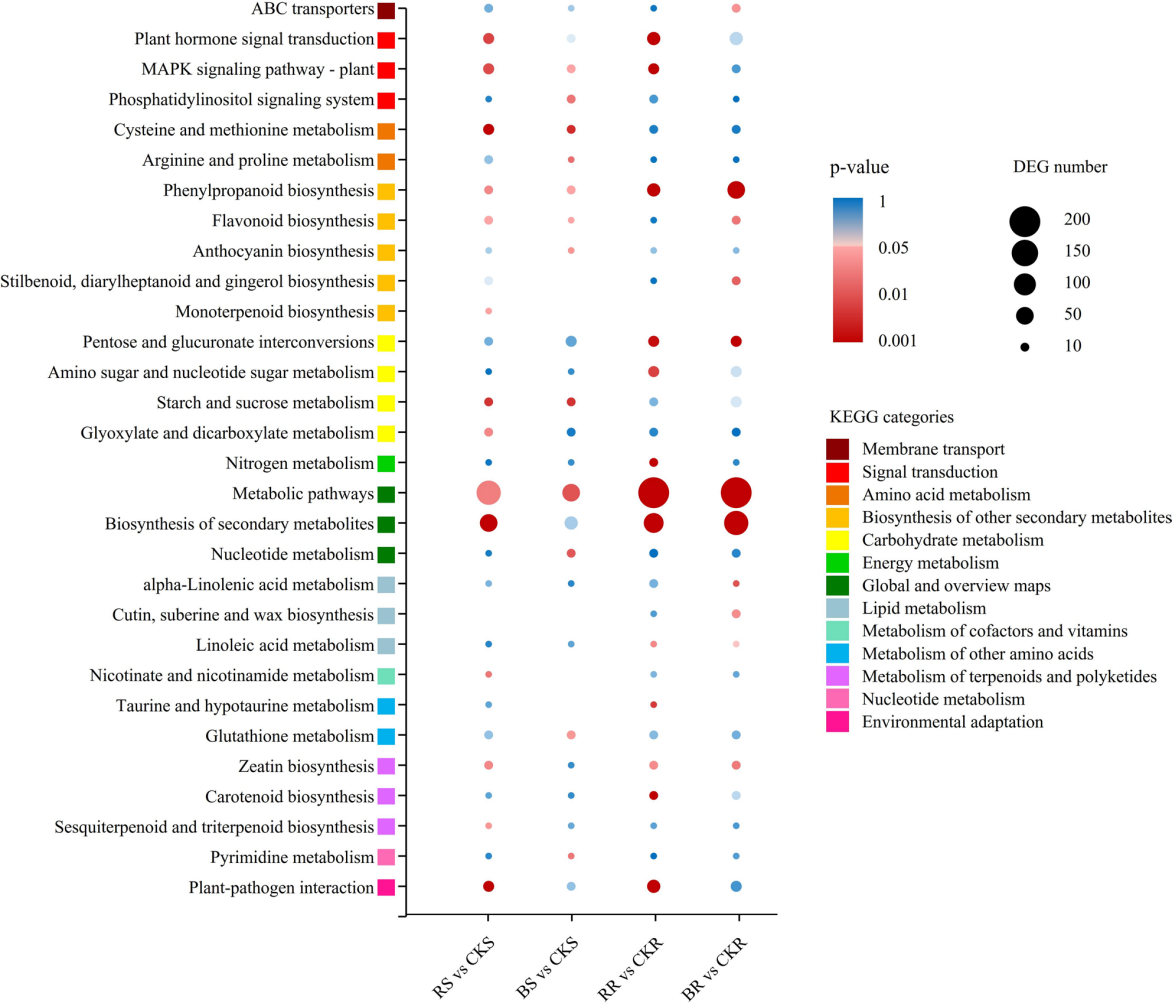
KEGG pathway enrichment analysis of DEGs. Bubbles are colored by *p*-value as indicated in the color legend. Bubble size represents the number of DEGs involved in each KEGG pathway. The color of the square adjacent to each KEGG pathway denotes the corresponding KEGG category. *Abbreviations*: *CKS* Shoots under white light treatment, *RS* Shoots under monochromatic red light treatment, *BS* Shoots under monochromatic blue light treatment, *CKR* Roots under white light treatment, *RR* Roots under monochromatic red light treatment, *BR* Roots under monochromatic blue light treatment

Furthermore, KEGG pathway analysis revealed that the majority of enriched pathways were associated with metabolism, including “alpha-linolenic acid metabolism”, “phenylpropanoid biosynthesis”, “arginine and proline metabolism”, “starch and sucrose metabolism”, and “nucleotide metabolism” (Table S5, Fig. 4). Notably, several KEGG pathways in BR and RR were related to hormone signaling and biosynthesis (Table S5A, B, Fig. 4), such as “Plant hormone signal transduction”, “alpha-linolenic acid metabolism”, “zeatin biosynthesis”, and “carotenoid biosynthesis”, suggesting that the biosynthesis of endogenous hormones in tobacco roots is likely regulated by blue and red light. Additionally, “nitrogen metabolism” was enriched in both BR and RR (Table S5A, B, Fig. 4), indicating that nitrogen metabolism in roots may also be affected by monochromatic light.

### Effects of monochromatic light on root nitrogen metabolism genes

**Fig. 5:**
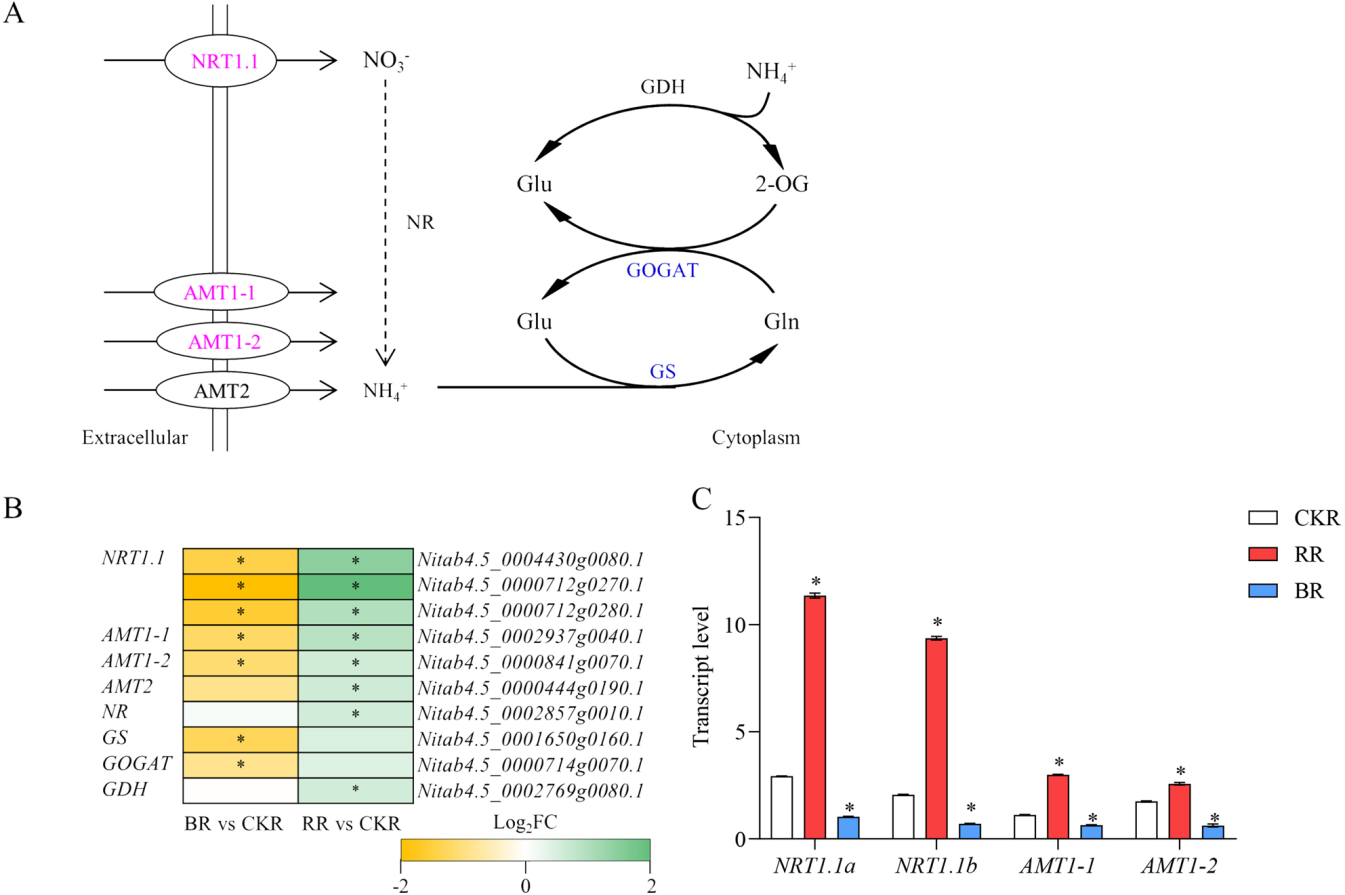
Effects of monochromatic light treatments on N metabolism-related genes.** A** DEGs involved in the N metabolism pathway. Genes highlighted in blue were significantly regulated under blue light, while those highlighted in magenta were significantly regulated under both red and blue light. Solid arrows indicate specific steps or reaction processes in metabolic flux, and dotted arrows indicate multiple steps or reaction processes in metabolic flux. *Abbreviations*: *AMT* Ammonium transporter, *GDH* Glutamate dehydrogenase, *GOGAT* Glutamate synthase, *GS* Glutamine synthetase, *NR* Nitrate reductase, *NRT* Nitrate transporter. **B** Fold changes in the expression of N metabolism genes in response to monochromatic blue and red light treatments. * indicates significant differences (as specified in Materials and Methods). **C** Transcript levels of *NRT1.1a* (Nitab4.5_0004430g0080.1), *NRT1.1b* (Nitab4.5_0000712g0270.1), *AMT1-1* (Nitab4.5_0002937g0040.1) and *AMT1-2* (Nitab4.5_0000841g0070.1) were analyzed relative to that of *NtL25* (GenBank: L18908.1), a stable reference gene. * represents significant difference compared with white light treatment (*P* < 0.01, Student’s *t*-test). *Abbreviations*: *CKR* Roots under white light treatment, *RR* Roots under monochromatic red light treatment, *BR* Roots under monochromatic blue light treatment

Key genes involved in N uptake and assimilation were analyzed (Fig. 5A). Three nitrate transporter (NTR) genes encoding *NRT1.1* were significantly regulated by monochromatic light, with red light inducing and blue light suppressing their expression, respectively (Fig. 5B). Ammonium transporter (AMT) genes, including *AMT1-1*, *AMT1-2*, and *AMT2*, were upregulated by red light but downregulated by blue light (Fig. 5B). Monochromatic light also modulated N assimilation, although red and blue light exerted distinct effects on genes encoding key enzymes. Red light positively regulated nitrate reductase (*NR*), glutamine synthetase (*GS*), glutamate dehydrogenase (*GDH*), and glutamate synthase (*GOGAT*) genes, though only the regulation of *NR* and *GDH* genes reached a significant level (Fig. 5B). In contrast, blue light negatively regulated *GS* and *GOGAT* but had no apparent effect on *NR* and *GDH* (Fig. 5B). Further RT-qPCR experiments were conducted for validation. The results indicated that *NRT1.1a*, *NRT1.1b*, *AMT1-1* and *AMT1-2* expression was upregulated by red light stimulation and downregulated by blue light inhibition (Fig. 5C). Collectively, genes associated with both N uptake and assimilation in tobacco roots were significantly affected by monochromatic light. To validate this, we performed a correlation analysis, which revealed a strong positive correlation between the expression levels of key genes (*NRT1.1*,* AMT*,* GS* and *GOGAT*) and the total nitrogen content in the roots (Pearson’s *r* > 0.85, *p* < 0.05), directly linking the transcriptional changes to the observed physiological phenotype. These results indicate that red light likely promotes N metabolism by stimulating nitrogen uptake and assimilation, while blue light probably suppresses these processes.

### Effects of monochromatic light on root potassium metabolism genes

**Fig. 6:**
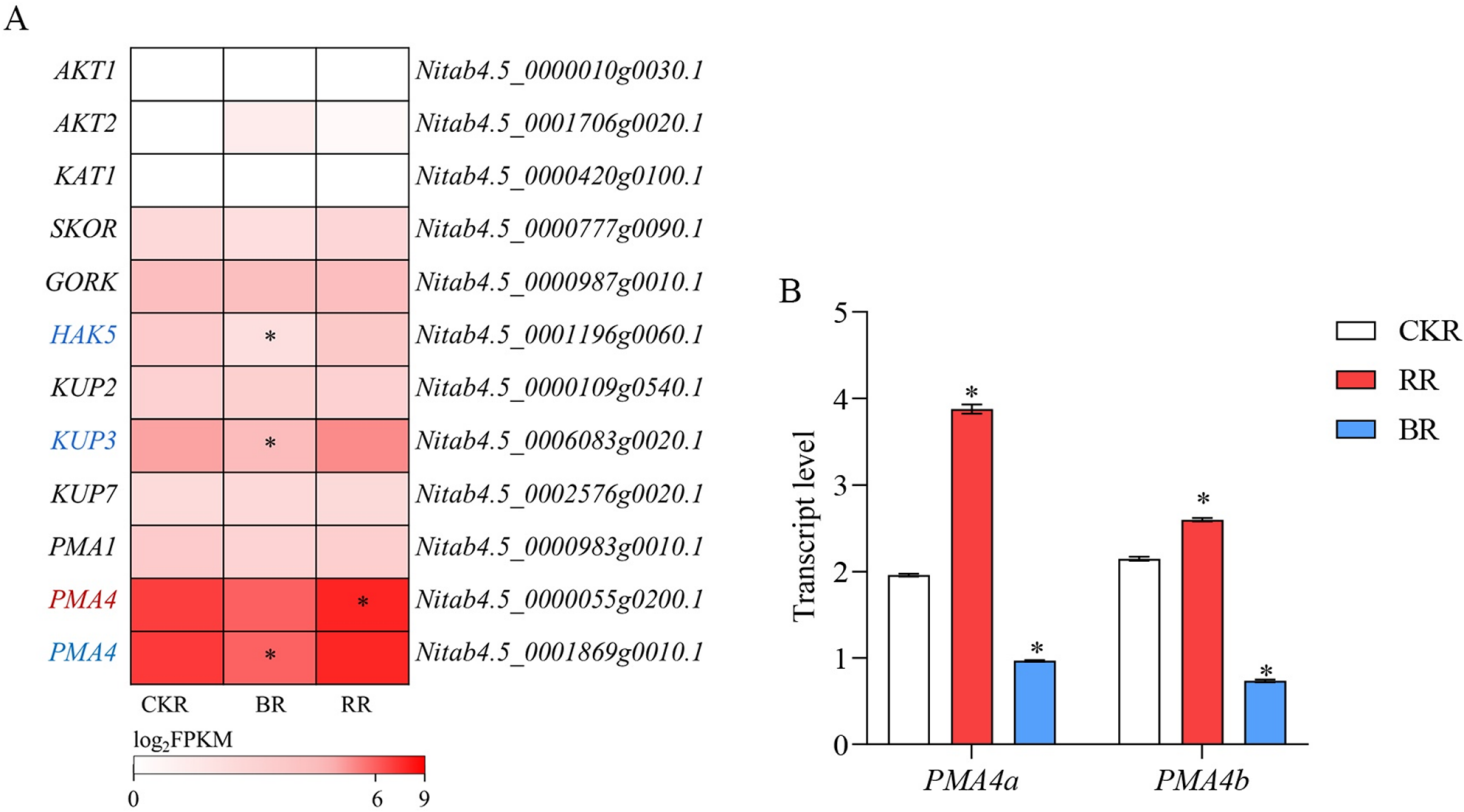
Effects of monochromatic light treatments on K⁺ uptake-related genes.** A** Fold changes in the expression of K⁺ uptake-related genes in response to monochromatic blue and red light treatments. * indicates significant differences compared with the white light treatment (as specified in Materials and Methods). **B** Transcript levels of *PMA4a* (Nitab4.5_0000055g0200.1) and *PMA4b* (Nitab4.5_0001869g0010.1), were analyzed relative to that of *NtL25* (GenBank: L18908.1), a stable reference gene. * represents significant difference compared with white light treatment (*P* < 0.01, Student’s *t*-test). *Abbreviations*: *CKR* Roots under white light treatment, *RR* Roots under monochromatic red light treatment, *BR* Roots under monochromatic blue light treatment

To investigate the effect of monochromatic light on K⁺ uptake, genes encoding K⁺ transport proteins were analyzed. The expression patterns of K⁺ channel genes (*AKT1*, *AKT2*, *KAT1*, *SORK* and *GORK*) and K⁺ transporter genes (*HAK5*, *KUP2*, *KUP3*, and *KUP7*) are presented in Fig. 6. The expression levels of *AKT1*, *AKT2* and *KAT1* in roots were extremely low (maximum FPKM < 5), precluding their use in subsequent analyses (Fig. 6A). Except for *HAK5* and *KUP3*, which were negatively regulated by blue light, other K⁺ transport genes were not affected by monochromatic light (Fig. 6A). Given that PM H^+^-ATPases also modulate K⁺ uptake [39], the transcription levels of PM H⁺-ATPase genes were determined. Three PM H⁺-ATPase genes were expressed in roots. *PAM1* was not significantly regulated by either monochromatic red or blue light (Fig. 6A). One *PAM4* homolog was significantly induced by red light, while the other was significantly downregulated under blue light (Fig. 6A). Further RT-qPCR experiments were conducted for validation. The results indicated that *PAM4* expression was upregulated by red light stimulation and downregulated by blue light inhibition (Fig. 6B).

### Key transcription factors involved in root responses to red and blue light

**Fig. 7:**
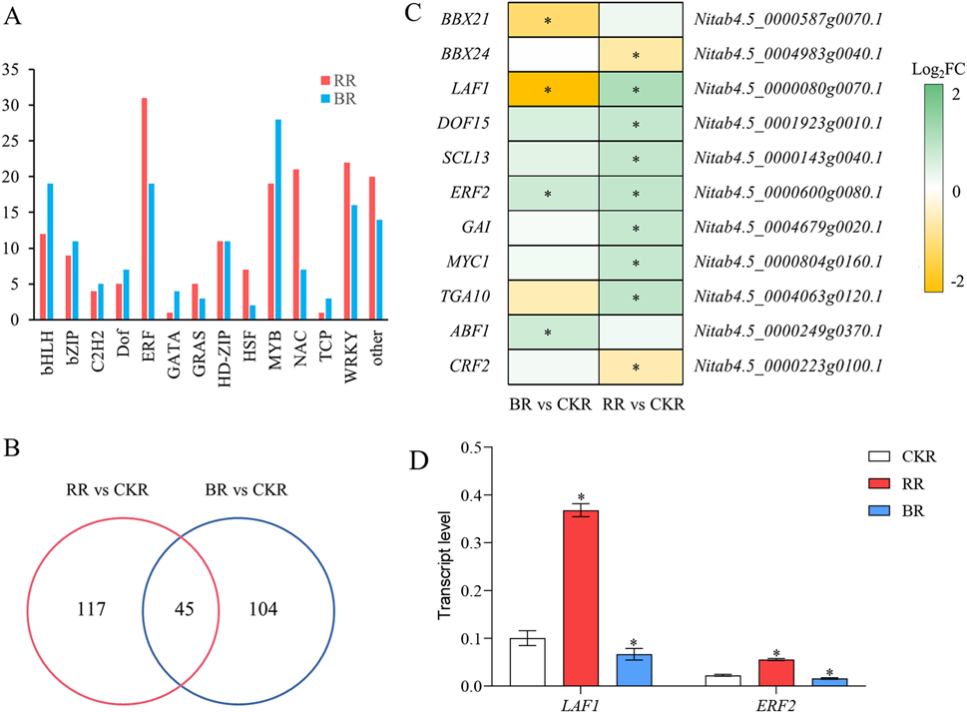
Effects of monochromatic light treatments on the expression of TFs.** A** Categories of TFs regulated by monochromatic light. **B** Venn diagram of differentially expressed TFs. **C** Fold changes in the expression of differentially expressed TFs in response to monochromatic light treatments. * indicates significant differences compared with the white light treatment (as specified in Materials and Methods). **D** Transcript levels of *LAF1* (Nitab4.5_0000080g0070.1) and *ERF2* (Nitab4.5_0000600g0080.1) were analyzed relative to that of *NtL25* (GenBank: L18908.1), a stable reference gene. * represents significant difference compared with white light treatment (*P* < 0.01, Student’s *t*-test). *Abbreviations*: *CKR* Roots under white light treatment, *RR* Roots under monochromatic red light treatment, *BR* Roots under monochromatic blue light treatment

Compared with the white light control, 168 and 149 transcription factors (TFs) were differentially expressed in roots under monochromatic red light and monochromatic blue light treatments, respectively (Fig. 7A). Under red light treatment, the ERF family was the most abundant, followed by the WRKY, NAC, and MYB families. In contrast, the top three TF families under blue light treatment were the MYB, ERF, and bHLH families. A total of 45 TFs were co-regulated by both red and blue light treatments (Fig. 7B). The *BBX21* and *BBX24* genes were significantly regulated by blue light and red light, respectively. The *LAF1* gene (MYB family) was significantly upregulated in RR but downregulated in BR. Additionally, the *DOF15* gene (DOF family) and *SCL13* gene (GRAS family) were significantly induced by red light. All five genes are involved in light signal transduction. Furthermore, numerous phytohormone-responsive TFs were significantly regulated by monochromatic light (Fig. 7C). For instance, the *GAI* gene (GRAS family), *MYC1* gene (MYC family), and *TGA10* gene (TGA family) were induced by red light, while the ABA-responsive element binding factor *ABF1* was induced by blue light. Notably, the *CRF2* gene (ERF family) was significantly suppressed in RR. Further RT-qPCR experiments were conducted for validation. The results indicated that *LAF* and *ERF2* expression was upregulated by red light stimulation and downregulated by blue light inhibition (Fig. 7D).

## Discussion

Physiological analyses revealed that light quality significantly affects plant growth (Fig. 1). However, no notable difference in leaf number was observed among white, red, and blue light treatments (Fig. S1), indicating that seedlings under these light conditions were at the same developmental stage. Therefore, the influence of development-related genes [40] on transcriptomic analysis can be excluded.

### Monochromatic light significantly influenced tobacco seedling growth

Roots exhibited greater sensitivity to monochromatic light than shoots, as evidenced by the higher number of DEGs in roots compared with shoots (Fig. 2B-C). In shoots, the number of DEGs under red light treatment was approximately three times that under blue light (1459 vs. 459), consistent with the observation that red light has a more pronounced impact on shoot biomass (Fig. 1C). In roots, the number of DEGs was comparable between red and blue light treatments (2124 vs. 2088), with only 541 DEGs shared by both light treatments. Monochromatic red and blue light exerted opposite effects on root growth (Fig. 1B). Interestingly, roots showed a greater transcriptomic response to light quality than shoots, with a higher number of DEGs. This heightened sensitivity in a non-photosynthetic organ could be attributed to several potential mechanisms. Shoots, as the primary light-perceiving organs, may transmit long-distance signals, such as auxin, cytokinin, or sucrose, to the roots, thereby modulating gene expression. It is also possible that roots possess their own photoreceptors that can perceive light transmitted through the soil or stem, although this signaling would be weaker than in shoots. The complex interplay of these systemic signals likely leads to the extensive transcriptional reprogramming observed in the roots.

### Red and blue light exert opposite effects on N metabolism in roots

Both transcriptomic and physiological data indicated that red light promoted N metabolism, whereas blue light inhibited it. KEGG pathway enrichment analysis revealed that both red and blue light treatments modulated N metabolism in tobacco roots (Fig. 4). This finding was further supported by significant alterations in total N content in the roots under different light treatments (Fig. 1J). N metabolism primarily involves N uptake and assimilation processes (Fig. 5A).

Plants acquire N from the soil primarily in the forms of nitrate (NO_3_⁻) and ammonium (NH_4_⁺), which are facilitated by NRTs and AMTs, respectively [41, 42]. Nitrogen assimilation initiates with the reduction of NO_3_⁻ to NH_4_^+^—a process sequentially catalyzed by NR and nitrite reductase (NiR) [43, 44]—and culminates in the incorporation of NH_4_^+^ into amino acids via the GS/GOGAT cycle [45]. NR, NiR, GS, and GOGAT are key enzymes involved in N assimilation. GDH, an enzyme catalyzing the reversible formation of glutamate from NH_4_^+^ and 2-oxoglutarate, also plays a prominent role in N assimilation [46].

Under blue light treatment, the expression of NO_3_⁻ transporter genes (*NRT1.1*) and NH_4_^+^ transporter genes (*AMT1-1* and *AMT1-2*) was downregulated (Fig. 5B, C), indicating a reduction in N uptake [47, 48]. Conversely, red light treatment induced the expression of *NRT1.1*, *AMT1-1*, *AMT1-2*, and *AMT2*, suggesting that red light enhances N uptake [49–52]. The GS/GOGAT cycle is the primary pathway for N assimilation in crops [45, 53]; thus, the observed suppression of *GS* and *GOGAT* indicated that blue light negatively regulated N assimilation. NR, which catalyzes the initial step of nitrate assimilation and is upregulated by NO_3_⁻, functions as a rate-limiting enzyme in NO_3_⁻ assimilation [43]. Red light positively regulated *NR*, suggesting that NO_3_⁻ assimilation was enhanced by red light, rather than merely NO_3_⁻ uptake. GDH expression was also induced by red light, likely facilitating the removal of excess NH_4_⁺ generated from increased NO_3_⁻ reduction and NH_4_⁺ uptake, thereby preventing the accumulation of toxic NH_4_⁺ levels within cells [45, 54]. Collectively, these results indicate that red light likely promotes N metabolism by enhancing N uptake and assimilation, while blue light probably suppresses N uptake and assimilation, thereby inhibiting N metabolism.

### Red and blue light exert opposite effects on K uptake in roots

Similar to N, K⁺ is an indispensable nutrient for plant growth, and the uptake of N and K⁺ in plants is subject to co-regulation [55, 56]. The activation of PM H^+^-ATPase under red light, which energizes the plasma membrane, provides the driving force for both K^+^ channels and some N transporters, offering a mechanistic basis for their coordinated enhancement. K⁺ transport proteins involved in transmembrane K⁺ transport mainly belong to the Shaker K⁺ channel family (AKT1, AKT2, KAT1, KAT2, SORK, and GORK) and the HAK/KUP/KT K⁺ transporter family (HAK5, KUP2, KUP3, and KUP7) [26]. AKT1, SKOR, and AKT2 are responsible for K⁺ uptake into root cells, loading into xylem sap, and transport through the phloem, respectively [57–60]. KAT1, KAT2, and GORK regulate stomatal movements by controlling K⁺ fluxes in guard cells [61, 62]. Several KUP/HAK/KT transporters, including HAK5, KUP2, KUP3, and KUP7, were expressed in roots and contribute to K⁺ uptake [26, 63]. PM H⁺-ATPases also modulate K⁺ uptake [39].

Increased PM H⁺-ATPase activity leads to H⁺ extrusion, causing extracellular acidification [64], which in turn activates K⁺ channels and promotes K⁺ transport and uptake [65–67]. PM H⁺-ATPase activity is positively associated with K⁺ uptake efficiency [29, 67]. Compared with the control, red light significantly increased root K⁺ content (Fig. 1K), yet none of the K⁺ transport protein genes were significantly regulated (Fig. 6). Given that PM H⁺-ATPases can regulate K⁺ uptake efficiency, the expression and activity of PM H⁺-ATPases were determined to clarify the mechanism underlying enhanced K⁺ uptake. As expected, PM H⁺-ATPase activity was significantly increased (Fig. 1L), and the expression of PM H⁺-ATPase genes was significantly upregulated by red light (Fig. 6). In contrast to red light, blue light significantly downregulated *HAK5* and *KUP3* genes. Meanwhile, both the transcription and activity of PM H⁺-ATPases were suppressed by blue light (Figs. 1L and 6). Collectively, red light likely enhances K⁺ uptake by activating PM H⁺-ATPases, while blue light probably reduces K⁺ uptake by suppressing the transcription of K⁺ transporters and the activity of PM H⁺-ATPases. This activation could be regulated at both transcriptional and post-translational levels. Our data show upregulation of a *PAM4* gene, but light-induced hormonal changes (e.g., auxin) are also known to trigger rapid, non-transcriptional activation of PM H^+^-ATPases through phosphorylation, providing another layer of control.

### Monochromatic light regulate seedling growth via TF-mediated pathways

A total of 162 and 149 differentially expressed TFs were identified in RR and BR, respectively, with only 45 TFs shared between the tissue comparisons (Fig. 7A-B). These results suggest that red and blue light likely regulate distinct biological processes. Among the identified differentially expressed TFs, BBX21, BBX24, LAF1, DOF15, and SCL13 are known to be involved in light signal transduction [7, 10, 68, 69]. BBX21, BBX24, and LAF1 function as regulators of seedling photomorphogenesis—particularly in root development—by interacting with COP1 and HY5, the major light signaling regulators in plants [68–71]. DOF15 and SCL13 participate in the transmission of phytochrome signals and regulate photomorphogenesis [72, 73]. These TFs were significantly regulated by red and blue light (Fig. 7C, D), underscoring the critical role of transcriptional regulation in light signal transduction in tobacco roots.

Numerous studies have reported that light quality modulates plant hormone levels [11, 12]. Herein, key TFs involved in plant hormone signal transduction were regulated by monochromatic light. For instance, ERF2, GAI, MYC1, TGA10, CRF2, and ABF1—critical regulators of ethylene (ETH), gibberellin (GA), jasmonic acid (JA), salicylic acid (SA), cytokinin (CT), and abscisic acid (ABA) signaling pathways, respectively [74–79]—exhibited differential regulation in response to red and blue light (Fig. 7C, D). KEGG pathway analysis revealed that pathways associated with hormone biosynthesis, such as alpha-linolenic acid metabolism (JA biosynthesis), zeatin biosynthesis (CT biosynthesis), and carotenoid biosynthesis (ABA biosynthesis), were enriched in DEGs from the RR/CKR and BR/CKR comparisons (Fig. 4, Table S5). Furthermore, the “plant hormone signal transduction” pathway was also enriched in both groups of DEGs. For instance, the induction of cytokinin response factor CRF2 under red light aligns with cytokinin’s known role in promoting cell division and biomass accumulation, consistent with the increased root and shoot dry weight observed. Conversely, the induction of ABA-responsive factor ABF1 by blue light could contribute to the observed growth inhibition, as ABA often acts as a growth antagonist. Collectively, these results indicate that monochromatic light likely regulates the biosynthesis and signaling of plant hormones through TF-mediated pathways.

### Prospects for future research

The findings of this study provide valuable insights into the effects of monochromatic red and blue light on the growth and nutrient uptake of tobacco seedlings. However, it is important to acknowledge the study’s limitations and consider the implications for future research. This experiment was conducted under controlled environmental conditions, which may not fully simulate the complex scenarios encountered in natural or large-scale agricultural settings. Therefore, further studies are warranted to evaluate the generalizability of our findings to other plant species and diverse environmental conditions. Additionally, integrating multi-omics data with physiological measurements will be crucial for developing a comprehensive understanding of light-mediated regulation of plant growth and nutrient dynamics. Functional validation approaches, such as mutant screening, gene knockout, and overexpression assays, are further essential to validate the proposed hypotheses. For long-distance mobile mRNAs transported from shoots to roots under red and blue light, grafting experiments may offer insights into the functions of specific genes in light responses and nutrient dynamics. Notably, the roles of phytochromes and cryptochromes in roots remain understudied, with most research focusing on shoot organs like leaves and stems. Future studies could explore their functions in root growth and environmental adaptation, offering new insights for optimizing cultivation via light regulation. Ultimately, these findings should inform the development of strategies to optimize light quality in agricultural practices, potentially leading to improved crop yield and quality.

From an agricultural perspective, these findings have practical implications. For example, in controlled environment agriculture like vertical farms, supplementing red light could be a strategy to enhance nutrient use efficiency, particularly for nitrogen and potassium, potentially reducing fertilizer costs and environmental runoff. Conversely, a higher proportion of blue light might be used to produce more compact seedlings for easier transplanting. Fine-tuning light spectrum could become a powerful tool to optimize crop growth, yield, and quality.

## Conclusion

This study elucidates the distinct effects of monochromatic red and blue light on the growth and nutrient uptake of tobacco seedlings, demonstrating that red light promotes biomass accumulation and nutrient assimilation, whereas blue light exerts the opposite effect. Through transcriptomic analysis, we identified key genes and transcription factors regulated by light quality, suggesting the existence of a complex regulatory network modulating plant development. Despite being preliminary, our findings provide meaningful insights for the optimization of agricultural practices and further our knowledge of light-regulated plant growth and development.

## Supplementary Information

Supplementary Material 1.

## Data Availability

The raw sequence reads have been deposited in the NCBI sequence read archive (SRA) database under the accession number (Bioproject ID: PRJNA983570). All other data generated or analyzed during this study are included in this published article.
