# Supplementary material for: Transcriptomic analysis reveals light quality response and systemic nutrient allocation under monochromatic light environments in Nicotiana tabacum
Source: BMC Plant Biol. 2025 Dec 20;25:1750. doi: 10.1186/s12870-025-07974-w (PMC12750675; doi:10.1186/s12870-025-07974-w)
Supplement: Supplementary file 1 — Supplementary Material 1. [file 12870_2025_7974_MOESM1_ESM.zip › Supplementary Materials-revised-V3/Supplemental Figure..docx]

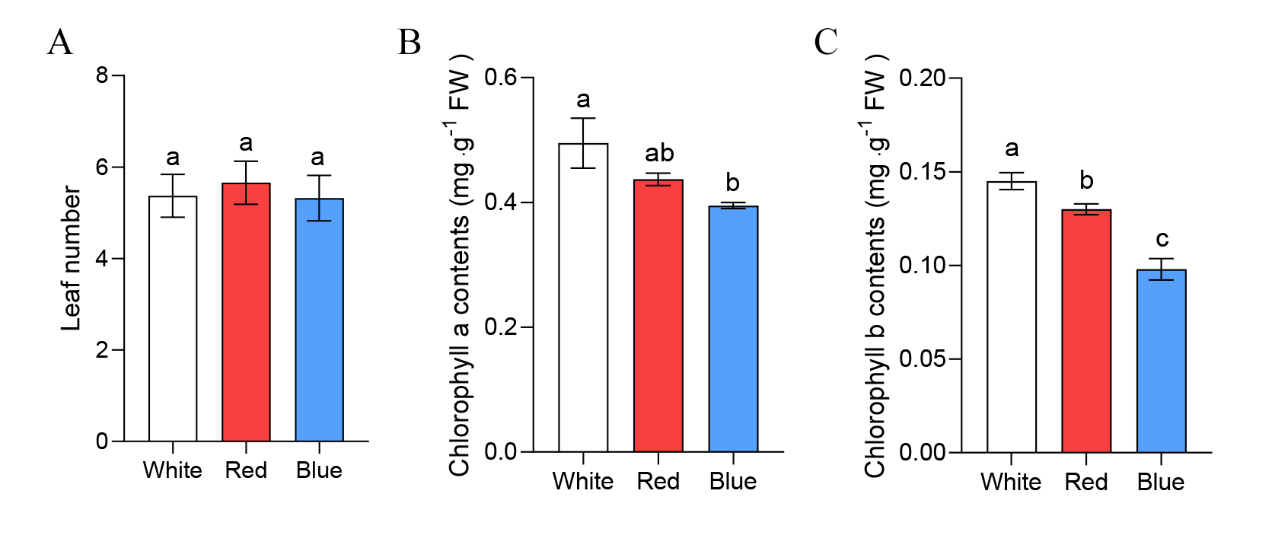


Fig. S1. Physiological responses of tobacco seedlings to monochromatic light treatments. (A) The leaf number of tobacco seedlings treated with different light quality. (B, C) Chlorophyll a content (B) and Chlorophyll b content (C) of tobacco seedlings treated with different light quality. *N*=5, biological repetition. Different letters indicate significant differences between the data. Ordinary one-way ANOVA Tukey's multiple comparisons test, *p* < 0.05.


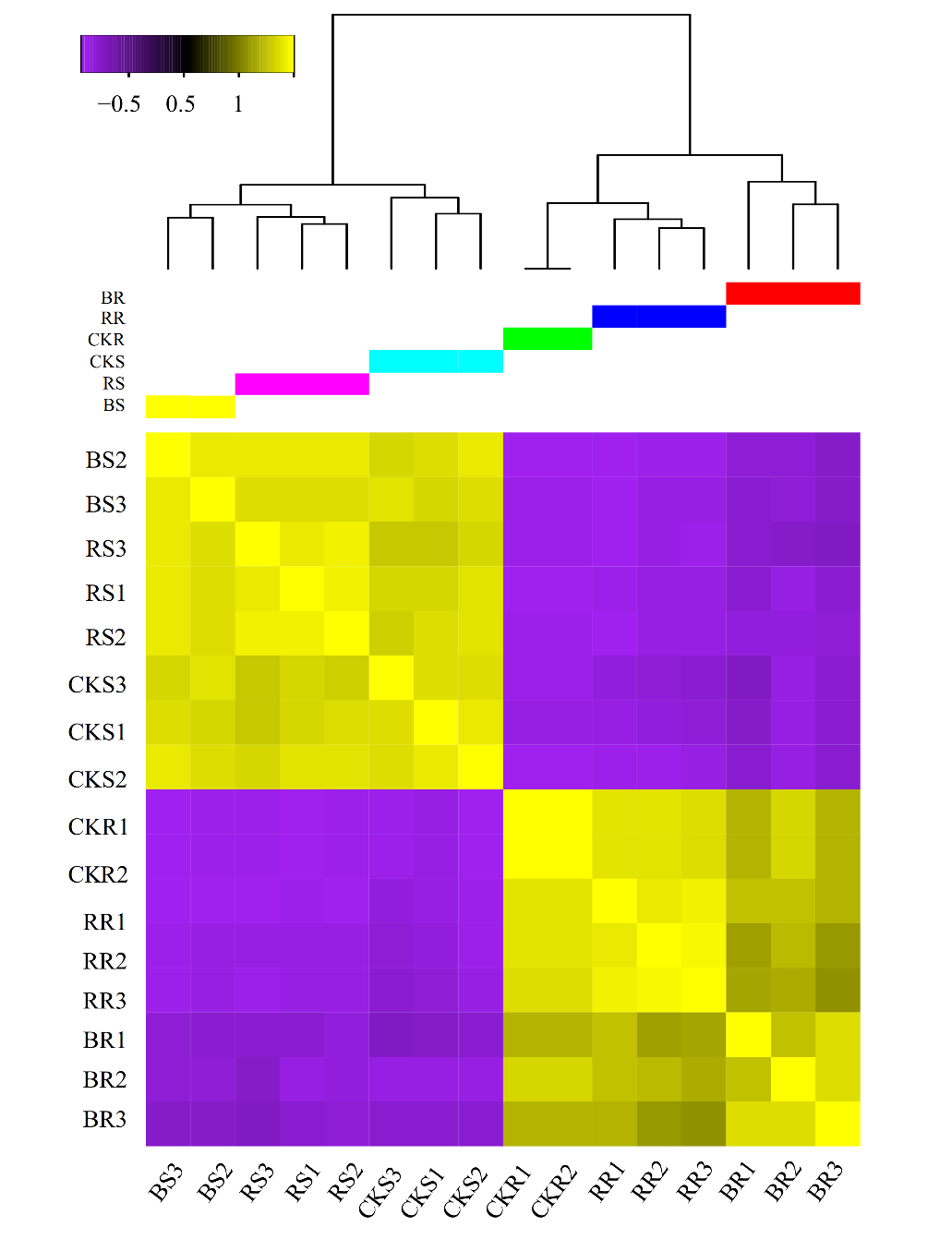


Fig. S2. Correlation matrix heatmap calculated from pairwise comparisons among samples. Abbreviations: CKS, shoots under white light treatment; RS, shoots under monochromatic red light treatment; BS, shoots under monochromatic blue light treatment; CKR, roots under white light treatment; RR, roots under monochromatic red light treatment; BR, roots under monochromatic blue light treatment.
